# The effect of maternal body mass index on embryo division timings in women undergoing in vitro fertilization

**DOI:** 10.1016/j.xfre.2022.10.004

**Published:** 2022-10-20

**Authors:** Theresa Piquette, Robert T. Rydze, Amy Pan, Jayme Bosler, Amy Granlund, Kate D. Schoyer

**Affiliations:** a Division of Reproductive Endocrinology and Infertility, Department of Obstetrics and Gynecology, Medical College of Wisconsin, Milwaukee, Wisconsin; b Division of Quantitative Health Sciences, Department of Pediatrics, Medical College of Wisconsin, Milwaukee, Wisconsin; c Reproductive Medicine Center, Froedtert Hospital, North Hills Health Center, Menomonee Falls, Wisconsin

**Keywords:** obesity, in vitro fertilization, time-lapse, embryoscope, morphokinetic

## Abstract

**Objective:**

To measure the impact of maternal body mass index (BMI) on the morphokinetics of embryo development as monitored by a time-lapse system.

**Design:**

A retrospective chart review of in vitro fertilization (IVF) cycles from September 2016 to January 2019.

**Setting:**

Academic IVF practice.

**Patient(s):**

Patients <age 38 years undergoing IVF with their own gametes.

**Intervention(s):**

Not applicable.

**Main outcome measure(s):**

The primary outcome was to compare embryo division timings between morbidly obese, obese, overweight, and normal-weight patients. A multilevel mixed effects model was performed to investigate the relationships between BMI categories and embryo division timings. Log or square transformation were used to improve fit.

**Result(s):**

A total of 366 patients met inclusion criteria, yielding 4,475 embryos: 1,948 embryos from 162 normal-weight women (BMI 18.5–24.9), 1,242 embryos from 96 overweight women (BMI 25.0–29.9), 1,119 embryos from 91 obese women (BMI 30.0–39.9), and 166 embryos from 17 morbidly obese women (BMI ≥40). There were no differences in age, Antimüllerian hormone, or IVF cycle outcomes among the different BMI categories. When comparing embryo division timings based on BMI, controlling for covariates, embryos from obese patients had a shorter time to division to 2 cell embryo (T2) than normal-weight patients. When analyzing BMI as a continuous variable, there was no significant relationship between BMI and embryo division timing.

**Conclusion(s):**

Early embryo divisions were accelerated in only certain categories of obesity. This suggests a more complex mechanism for the effect of obesity on embryo development that may not be perceptible through the assessment of cell division timing events.

Obesity is an epidemic with rates increasing in the last few decades; at least 50% of reproductive-age women are overweight or obese (1). Obesity has detrimental effects on fertility, including anovulation, longer time to conception, and miscarriage (2, 3, 4). Unfortunately, these detrimental effects on spontaneous fertility also translate into reduced success with assisted reproductive technologies such as IVF, resulting in a decreased number of oocytes retrieved, impaired embryo quality, reduced implantation rates, increased miscarriage rates, and overall diminished live birth rates (3, 5, 6, 7, 8, 9, 10, 11, 12, 13, 14).

Despite these demonstrated effects in clinical outcomes, results from traditional methods of evaluation of embryo quality, including morphological grade, do not consistently differ based on BMI, suggesting that morphology alone may not reflect the developmental potential of embryos conceived with oocytes from obese women undergoing IVF (11, 15, 16, 17).

Given the availability of time-lapse monitoring systems that track the specific division patterns and kinetics of embryos, it is now possible to observe and study the patterned and dynamic progression of embryos as they develop. Careful observation of these coordinated series of timed cell divisions may provide further insight into the effects of obesity on the observed decrease in fertility (18, 19). These morphokinetic parameters include the time required to divide into a given number of cells, cleavage patterns, and cell cycle lengths. Particular criteria based on these measures have been used to identify embryos with either ideal or aberrant morphokinetics to select embryos with the best implantation potential (20, 21, 22, 23). However, factors affecting the timing of these divisions are not well understood, including the effect of obesity (24, 25, 26).

Bellver et al. (27) compared embryos from normal weight, overweight, infertile, and fertile women to determine that infertile women, regardless of obesity or normal weight, produced embryos that had significantly slower division timings throughout development compared with oocyte donors. However, because of an underpowered sample size and obese BMI limited by a mean of 32kg/m^2^, they could not conclude an association between obesity and embryokinetics. This limits applicability to a population primarily comprised of overweight and obese reproductively aged women presenting for assisted reproductive technologies at clinics that provide treatment to women with higher BMI.

More compelling evidence for an association between obesity and embryo division comes from Finger et al. (28) who, using a mouse model of diet-induced obesity, demonstrated prolongation in the division timing of embryos dependent on parental BMI. Specifically, they noted a delay in division timing between 1 to 6 hours from the second cell division to the formation of a blastocyst, respectively, comparing obese-parented embryos to normal-weight parented mouse embryos. A similar delay may exist in human embryos leading to alterations in embryo division timing based on increased BMI, which may ultimately highlight an aberrant mechanism for the reduction in fertility with increasing BMI for couples undergoing IVF.

To better elucidate the relationship between BMI and embryo morphokinetics, we aim to compare embryo morphokinetics in women undergoing their first cycle of IVF among patients of different BMI categories while controlling for several factors, including age, infertility diagnosis, Antimüllerian hormone (AMH) level, and smoking status. We hypothesize that embryo division timings, specifically T2 (time from one to two-cell embryo), will vary in a dose-dependent manner based on increasing maternal BMI.

## Materials and methods

This project was approved by the institutional review board of the Medical College of Wisconsin under the project “Optimizing Embryo Selection—Clinical Utility of the Embryoscope,” project Number # PRO00029347 with Amendment ID: AME00019854. Using the Froedtert Hospital Reproductive Medicine Center IVF patient database, we reviewed Vitrolife EmbryoScope Time-Lapse System data and patient medical records from women undergoing their first cycle of IVF from September 2016 to January 2019. We collected data on BMI at the time of egg retrieval as our main dependent variable. We also obtained patient demographics and covariates related to the patient's IVF cycle, such as age, gravity, parity, infertility diagnosis, chronic medical conditions, antagonist/agonist protocol, ampules of gonadotropins, days of stimulation, peak estradiol, number of oocytes retrieved/fertilized, the timing of division of embryo to 2 cell stage (T2), 3 cell stage (T3), four-cell stage (T4) and 5 cell stage (T5), formation of a blastocyst, morphologic grade of an embryo, day of the embryo at transfer, beta-human chorionic gonadotropin (hCG) or chemical pregnancy, fetal cardiac activity, and pregnancy outcome.

We attempted to minimize other factors that could negatively affect embryo development. Based on data from Liu et al, who reported that Embryoscope time-lapse imaging of polar body 2 extrusion was significantly delayed for women aged >38 years compared with those aged<35 years and between 35 and 38 years old, only patients aged <38 years were included in our study (29).

Exclusion criteria also encompassed those who had engaged in cigarette smoking within 6 months of the IVF cycle and who had a diagnosis of diminished ovarian reserve, based on AMH <1ng/mL, cycle day 2/3 serum Follicle Stimulating Hormone (FSH) >10 miu/mL, cycle day 2/3 serum estradiol >70 pg/mL) (30, 31). There were no patients with BMI >45 kg/m^2^, which is the upper BMI limit per our clinic policy with anesthesia for office-based sedation.

We also excluded patients with diminished ovarian reserve as there is data that AMH and antral follicle count are comparable predictors of oocytes retrieved and of the number of good-quality embryos available for transfer and freezing (30). Some investigators have found a positive correlation between AMH levels and oocyte and embryo quality (31, 32). Similarly, a retrospective cohort study of IVF cycles in the United States from 2009 to 2013 showed that cigarette smoking was associated with higher adjusted odds of IVF cycle cancellation with no embryo transfer (adjusted odds ratio [aOR]: 1.10; 95% confidence interval [CI]: 1.00–1.21) (33). Additionally, in a review of the effect of cigarette smoking on reproduction, Dechanet et al. describe how cigarette smoke exposure may induce meiotic spindle disturbances and disordered microfilaments in rodent models (34).

### Study Protocols

Regimens used during the study included either a “short” or a “long” protocol of down-regulation with a GnRH agonist followed by stimulation via gonadotropins or stimulation with gonadotropins followed by pituitary suppression with a GnRH antagonist.

Stimulation proceeded until at least 2 leading follicles reached 17–18 mm in size with appropriate estradiol levels.

Ovulation was triggered via hCG and/or leuprolide acetate based on clinician judgment. For GnRH antagonist protocols in which patients were deemed at high risk of ovarian hyperstimulation syndrome, ovulation was triggered by 2 mg leuprolide acetate administered with a low dose (1,500 IU) hCG or leuprolide acetate alone. Under conscious sedation, oocyte retrieval was performed 34–36 hours after hCG administration via transvaginal ultrasound-guided needle aspiration. The oocytes were then fertilized using intra-cytoplasmic semen injection (ICSI) or micro-insemination depending on patient preference, physician discretion, and/or male factor. Per protocol in our clinic, all cycles have at least a proportion of oocytes fertilized with ICSI. For luteal support, patients started either daily intramuscular progesterone (at 50 ng/mL) or vaginal gel the day after oocyte retrieval. In cases where leuprolide acetate and low-dose hCG trigger were used, the luteal phase was supported with 2 mg oral estradiol twice daily and one of the progesterone preparations. In cases at high risk for ovarian hyperstimulation syndrome, as deemed by clinic protocols (patient symptoms, more than 19 oocytes retrieved, peak estradiol levels >3,500 pg/ml, >19 growing follicles before hCG trigger), no luteal support was given and all embryos that were cryopreserved (35). During the study period, there was a trend toward an increased percentage of cycles in which embryos were cryopreserved upfront. Embryo transfer was performed 3–5 days later, depending on the number of embryos and embryo development. Our departmental policy is to perform a day 5 transfer if there are at least 5 normally fertilized embryos that develop well. One of 4 attending physicians performed embryo transfer, and all were done under ultrasound guidance used either the Sydney catheter or SureView Wallace catheter. Pregnancy tests for beta-hCG levels were performed 14 days after oocyte retrieval.

Metaphase 2 (M2) oocytes were fertilized using ICSI or conventional insemination. Oocytes were placed in group microdrop culture until fertilization check, which occurred 15–18 hours post ICSI. The COOK and MINC incubators were used post-ICSI culture until fertilization check. Mixed gas tanks with 6% carbon dioxide (CO_2_), 5% oxygen (O_2_), and the remaining balance of nitrogen, were used for the MINC incubators. Irvine Scientific (Fujifilm) Continuous Complete Culture NX (CSC-NX) was used for the full culture period. After fertilization was confirmed, the embryos were placed in the Embryoscope for continued culture until day 5/6 of development. Any blastocysts of adequate quality (minimum inner cell mass/trophectoderm grade of BB) according to a previously described grading system that were not transferred were cryopreserved on day 5 or 6 of culture using the Cryolock vitrification carrier system (36, 37). Blastocyst cryopreservation was performed by placing an embryo into 7.5% ethylene glycol and dimethyl sulfoxide for 9 min, followed by 15% ethylene glycol and dimethyl sulfoxide + 0.5 M sucrose for 60 seconds, and finally plunging the embryos directly into liquid nitrogen for storage. Embryoscope gas concentrations are set for 6% CO_2_, 5% O2.

### Evaluation of Time-lapse Images

All data generated from the EmbryoScope is analyzed using the EmbryoViewer software, which allows the viewing and analysis of embryos. No changes in embryo culture conditions were made throughout the study. Image acquisition was set for every 10 minute at 7 different focal planes for each embryo. Embryo image acquisition was performed with the software EmbryoViewer (Unisense FertiliTech, Aarhus, Denmark). Cleavage time was considered the moment when cell division was completed, and the 2 originating cells were completely segregated and invested by their respective cytoplasmic membranes. The definitions of the time points assessed in the study were as follows: T2, the time at which the embryo presented two separate and distinct cells; T3, the time at which a 3 blastomeres embryo was achieved; T4, the time at which a 4 blastomeres embryo was achieved; and T5, time at which a 5 blastomeres embryo was achieved.

### Outcome Measures

The primary outcome was embryo morphokinetics, for which parameters were the time points listed above. Embryo quality score, ongoing pregnancy, and the number of available blastocysts to transfer or cryopreserve were secondary outcomes. To generate the embryo quality score, the main parameters of assessment on day 3 were the degree of fragmentation, the number of blastomeres, and symmetry among blastomeres, whereas on day 5, the main parameters were degree of expansion, hatching status, inner cell mass, and trophectoderm quality (38). Clinical pregnancy was defined as the presence of fetal heartbeat at 6 to 12 weeks of gestation. Spontaneous abortion was defined as loss of clinical pregnancy before 20 weeks of gestation. Pregnancy rates were followed after fresh transfer only.

### Data Analysis

Median and interquartile range (IQR) were used to summarize the data. The chi-square test or Fisher’s exact test was used to examine the association between categorical variables, whereas the Kruskal-Wallis test was used for comparisons of continuous variables among BMI categories. A multilevel mixed effects model was employed to investigate the relationships between BMI categories or potential confounding factors (patient’s age, AMH, total number of oocytes retrieved, number of fertilized embryos, and number of blastocysts available for freeze/transfer) and the outcomes. A backward elimination method was used for model selection. The final model included BMI categories and any other significant factors (*P*<.05). Log transformation, or square transformation are used to improve fit. Logistic regression analysis was performed to test the effect of T2 on pregnancy results (hCG positive, FHM positive, live delivery). A value of *P* <.05 was considered statistically significant. Statistical software SAS V9.4 (SAS Institute Inc., Cary, NC) was used for analysis.

### Power Calculation

The primary outcome was T2, and the primary comparison was between normal-weight patients and overweight/obese patients. Based on preliminary data, each patient had an approximate average of 9 embryos. The intracluster correlation coefficient was set to be 0.01, whereas the coefficient of variation of the cluster sizes was 0.5. One hundred eighty-eight normal-weight patients with approximately 1,692 embryos and 94 overweight/obese patients with approximately 846 embryos were determined to have at least 80% power to detect a difference of 0.124 standard deviation (SD) with a significance level of 0.05.

## Results

A total of 366 patients met inclusion criteria, yielding 4,475 embryos: 1,948 embryos from 162 normal-weight women (median BMI=22.3 kg/m^2^), 1,242 embryos from 96 overweight women (median = 27.2), 1,119 embryos from 91 obese women (median = 34.0), and 166 embryos from 17 morbidly obese women (median = 43.5). There were no differences between BMI categories in age, AMH level, number of oocytes retrieved, or number of blastocysts available (Table 1). A total of 143 patients who met inclusion criteria underwent a fresh embryo transfer, with 52.8% having a positive pregnancy test and 40% having a clinical pregnancy.

**Table 1:** Patient demographics by BMI categories.

	Normal weight (N = 162)	Overweight (N = 96)	Obese (N = 91)	Morbidly obese (N = 17)	*P* value
BMI (kg/m^2^)	22.3 (20.9, 23.6)	27.2 (25.9, 28.6)	34.0 (31.9, 36.4)	43.5 (41.3, 44.3)	<.05
Age (y)	32.8 (30.9, 34.6)	32.7 (30.5, 35.4)	33.6 (31.1, 35.7)	33.3 (31.4, 36.7)	.38
AMH (ng/mL)	3.5 (2.2, 5.7)	3.4 (2.3, 5.5)	4.6 (2.4, 7.3)	4.8 (2.5, 6.4)	.33
Total number of oocytes retrieved	14 (9, 21)	13.5 (10, 19.5)	14 (10, 21)	12 (8, 17)	.54
Number of fertilized embryos	9 (6, 12)	8 (6, 12)	8 (5, 11)	8 (5, 10)	.40
Number of blastocysts	4 (2, 6)	4 (2, 6)	4 (2, 6)	3 (2, 4)	.22
Gravity (%)					.32
0	97 (60)	56 (58)	47 (52)	12 (70)	
1	33 (20)	18 (19)	28 (31)	4 (24)	
≥2	32 (20)	22 (23)	16 (17)	1 (6)	
Parity (%)					.8442
0	121 (75)	74 (77)	69 (76)	14 (82)	
1	31 (19)	17 (18)	20 (22)	3 (18)	
≥2	10 (6)	5 (5)	2 (2)	0 (0)	
Infertility Diagnosis n, (%)					.01
Anovulation	26 (16)	13 (14)	31 (34)	6 (35)	
Male factor	67 (41)	42 (44)	38 (42)	10 (59)	
Unexplained	44 (27)	20 (21)	11 (12)	1 (6)	
Tubal factor	18 (11)	12 (12)	10 (11)	0 (0)	
Endometriosis	6 (4)	7 (7)	1 (1)	0 (0)	
RPL	1 (1)	2 (2)	0 (0)	0 (0)	
Fertilization Method n, (%)					
ICSI	154 (95)	90 (94)	87 (96)	16 (94)	
Conventional	8 (5)	6 (6)	4 (4)	1 (6)	
IVF protocol					(.70)
Agonist	48 (30)	31 (32)	24 (26)	2 (12)	
Antagonist	114 (70)	65 (68)	67 (74)	15 (88)	

*Note:* Data were reported as median (IQR).

AMH = Antimüllerian hormone; BMI = body mass index; ICSI = intra-cytoplasmic semen injection; IVF = in vitro fertilization; RPL = recurrent pregnancy loss.

When comparing embryo division timings based on BMI, there were significant differences at the T2 time point. Embryos from obese patients had the shortest mean division timings (25.63 hours) compared with patients with normal weight (26.36 hours, *P*=.018), overweight (26.33 hours, *P*=.043) and morbidly obese (27.04 h, *P*=.032) patients (Table 2). This effect was persistent for all time points measured, including T3, T4, and T5 (*P*<.05).

**Table 2:** T2 division timing by BMI—univariate analysis.

BMI	Estimated T2 (Ln)	SE	T2 Time, (h)	95% CI
Normal weight^a^	3.27	0.007	26.4	26.0, 26.7
Overweight^a^	3.27	0.009	26.3	25.9, 26.8
Obese	3.24	0.009	25.6	25.2, 26.1
Morbidly obese^a^	3.30	0.023	27.0	25.9, 28.3

BMI = body mass index; CI = confidence interval; SE = standard error; T2 = the time at which the embryo presented 2 separate and distinct cells.

a *P*<.05 compared with obese patients.

Univariate analysis was performed to evaluate the effects of possible covariates, which revealed that patients whose embryos had faster division timings were more likely to have more blastocysts (*P*<.01). Similarly, the number of fertilized embryos was negatively associated with T2, T3, and T4 (*P*<.05). Interestingly, patient age, AMH level, and the total number of oocytes retrieved were not found to be significantly associated with the outcomes. Multivariable analysis using the backward elimination method showed that the number of blastocysts available and BMI categories were significant in the final model. The effect persisted where embryos from obese patients had the most rapid division timings compared with embryos from normal weight (*P*<.01), overweight (*P*<.05), and morbidly obese patients (*P*<.05) (Table 3). Again, this effect remained throughout our measured time points, including T3, T4, and T5 (*P*<.05) (Table 4). The ratio of blastocysts to fertilized embryos appears to be inversely related to T2, such that as the ratio increases (ie, with a higher proportion of blastocysts formed relative to the number of fertilized embryos), T2 decreases (*P*<.05).

**Table 3:** T2 division timing by BMI—multivariable analysis.

BMI	Estimated T2 (Ln)	SE	T2 Time, H	95% CI
Normal weight^a^	3.27	0.007	26.3	26.0, 26.7
Overweight^b^	3.27	0.009	26.2	25.7, 26.7
Obese	3.24	0.009	25.5	25.1, 26.0
Morbidly Obese^b^	3.29	0.023	26.8	25.6, 28.0

BMI = body mass index; CI = confidence interval; SE = standard error; T2 = the time at which the embryo presented 2 separate and distinct cells.

a *P*<.01 compared with obese patients.

b *P*<.05 compared with obese patients.

**Table 4:** T3 and T4 division timing by BMI—multivariable analysis.

BMI	T3 (95% CI)	T4 (95% CI)
Normal weight	36.9 (36.4, 37.3)^a^	38.5 (38.0, 39.0)^c^
Overweight	36.9 (36.3, 37.5)^b^	38.4 (37.8, 39.0)^b^
Obese	36.0 (35.4, 36.6)	37.4 (36.8, 38.0)
Morbidly Obese	37.7 (36.2, 39.2)^b^	39.1 (37.5, 40.7)^b^

BMI = body mass index; CI = confidence interval; T3 = time at which a 3 blastomeres embryo was achieved; T4 = time at which a 4 blastomeres embryo was achieved.

a *P*<.05 compared with obese patients.

b *P*<.05 compared with obese patients.

c *P*<.01 compared with obese patients.

However, when the data were analyzed using BMI as a continuous variable, there was no significant association between BMI and embryo division timing (*P*=.35). Timing to T2 was not affected by precycle IVF laboratory results, including day 3 estradiol, day 3 FSH, AMH levels, and antral follicle counts (*P*>.05).

Overall, the timing of T2 was found to be unrelated to infertility diagnosis when comparing anovulation, idiopathic, or another infertility diagnosis (*P*=.9). Distribution of infertility diagnosis was also analyzed between BMI categories, revealing a greater proportion of obese and morbidly obese patients carrying the diagnosis of anovulation (35% of morbidly obese and 34% of obese patients compared with 16% of normal-weight patients, *P*<.001). The BMI was found to differ significantly based on infertility diagnosis. Patients carrying a diagnosis of anovulation had a higher BMI than those with other infertility diagnoses (29.5 vs. 25.5 kg/m^2^) (*P*<.05).

Because of a possible confounding effect of anovulation, given the preponderance of this diagnosis among patients with higher BMI, data were analyzed excluding patients with a diagnosis of anovulation. Of the 366 patients, 272 did not carry a diagnosis of anovulation and were included for analysis. Within this patient group, 3,251 embryos were included in a multivariable analysis controlling for a covariate of the number of blastocysts available, which revealed no significant differences in embryo division timing based on maternal BMI (*P*>.05).

## Discussion

Contrary to our hypothesis, maternal BMI was found not to be associated with embryo morphokinetics in a dose-dependent manner. Unexpectedly, embryos from obese women had the most rapid division timings compared with all other BMI categories. These effects were persistent across time points from T2 through T5 and when controlling for covariates.

Interestingly, T2 also appeared to vary based on the outcomes of the stimulation cycle. Patients with more mature oocytes, more fertilized embryos, and more available blastocysts also demonstrated embryos with a shortened T2 interval. This pattern further supports the connection between more rapid embryo division timing and favorable IVF outcomes that have been well-described previously (20, 21, 22, 23, 24, 25, 26, 27, 39, 40).

The unexpected parabolic pattern of obesity’s relationship with embryo division timings, initially dose-dependent and then having the opposite effect as BMI continues to rise, was also described by Pinborg et al. (12) In their study of 487 infertile couples, they analyzed the effect of obesity on IVF outcomes and found an inverse U-shaped relationship with the overall number of developed embryos being highest for overweight and obese women, and lowest for underweight and morbidly obese women. This does not explain the alterations in division timings that we observed and suggests a more complicated relationship between BMI and embryo division timing.

Notably, overweight and obese women were more likely to carry a diagnosis of polycystic ovary syndrome (PCOS) or anovulation, yet the average BMI did not differ between these diagnosis groups. To control for a possible confounding effect of infertility diagnosis, we analyzed the data from patients without a diagnosis of PCOS or anovulation, which revealed no differences in division timings between the BMI groups.

Since we embarked on our study, more recent publications have examined the effect of obesity on embryo morphokinetics. Bartolacci et al. (15) studied the impact of obesity on 7,180 embryos derived from 1,528 IVF-ICSI cycles (15). After adjusting for age and infertility diagnosis, they found that T5 and time to reach 8 blastomeres (T8) were longer in obese women than in normal-weight women. However, the mean BMI in the 67 obese women was only 31.55 ± 1.49. In 2021, Barrie et al observed the morphokinetics of 2,376 embryos from 639 patients and found that T2 levels alone were associated with maternal BMI (41). They report the effect of BMI was not sustained throughout embryo development.

Our results and the results of these other studies suggest that obesity may have a complex interaction with embryo development that cannot be measured by embryo morphokinetics alone. Similarly to Finger et al. (28), Andreas et al. (42) used a mouse model for diet-induced obesity to monitor the effect of obesity across generations to note the metabolic differences among these embryos.They found that embryos from mice within the first-generation obesity group were less likely to develop to the blastocyst stage and had fewer cells. Further, they found that all generations, including the first, second, and third, had lower mitochondrial mass, adenosine 5′-triphosphate, and citrate than controls. They were able to reverse the effects of obesity through the enrichment of antioxidants into the culture medium.

These findings suggest a role of lipotoxicity, which is often described as a detrimental effect of obesity. Broughton and Moley describe a more severe infertility phenotype with combined obesity and PCOS, even when ovulatory, whether spontaneously or with assisted reproductive technology (5). Possible mediators of this effect are related to the increase in free fatty acids that cause a proinflammatory state leading to the release of cytokines and reactive oxygen species (43). These patterns observed in the mentioned studies and our study suggest that obesity and embryodynamics have a complex interplay.

Our study did not seek to investigate the relationship between paternal or combined parental obesity and embryodynamics, which may also contribute to our null results. We did not consistently collect data on the BMI of the sperm source and were not able to analyze this as a separate dependent variable, as was studied by Finger et al. (28). While with a mouse model, Finger et al. (28) found that combined parental obesity had a cumulative effect, human studies have not shown consistent results. Campbell et al. (44) performed a meta-analysis that suggested paternal obesity alone may lead to less favorable ART outcomes with reduced pregnancy and live birth rates. However, both Schliep et al. (16) and Colaci et al. (17), compared combined obese couples to normal-weight couples and failed to find such an effect with similarly rated embryo quality, pregnancy rate, and live birth rate across groups.

One limitation of our study was that data were collected from a single site, although we met the requirements of our power calculation and had a BMI distribution that corresponded with the general population. Our strict exclusion criteria may also limit generalizability to the population of women and couples seeking evaluation for infertility. A final limitation of this study was that it was not designed to elucidate the effect of BMI on pregnancy outcomes, a result that may be more relevant to patients in the clinical setting.

The strengths of our study include a larger sample size by comparison, especially of obese and morbidly obese women, to previous human studies analyzing time-lapse observation of embryo divisions (15, 27, 45). Bartolacci et al. (15) included 67 obese women and 286 embryos; Bellver et al. included 13 obese infertile women and 71 embryos; and Leary, Leese, and Sturmey included 29 overweight and obese women and 218 embryos.

Previous studies examining embryodynamics in obese patients also included patients with lower mean BMI levels: 28.37 in Leary et al; 33.6 in Bellver et al. (11); 32 in Bellver et al. (27); 26.5 in Colaci et al. (17); and 25.2 in Schliep et al. (16, 45). Only Shah et al. (7) analyzed class 2 (BMI 35.0–39.9) and class 3 (BMI 40+) obesity and Romanski et al analyzed class 3 and 4 (BMI 50+) specifically (14). However, both analyzed only IVF outcomes, including pregnancy rate, but did not examine embryo division timings. For our study, our large patient pool allowed us to have a distribution of BMI similar to the general population, including a broad range of BMI values that allowed subdivision into classes of obesity for further comparison, although we were not powered to detect differences between these subdivisions by obesity class individually.

Our statistical analysis attempted to control for the physiologic similarity within an individual patient by accounting for the variability of division timings of embryos that would be expected within a single patient embryo cohort. Although previous studies analyzed embryos as individual entities, it is physiologically and statistically true that multiple embryos from a single patient would be expected to behave more similarly than embryos compared from entirely different individuals.

Having collected additional data on covariates related to patient IVF cycles, we could better analyze and control for possible confounders affecting embryo division timings. We acknowledge the strict exclusion criteria, which may have skewed our results. However, the purpose of our exclusion criteria was to attempt to limit as many parameters that could potentially also impact as many factors that could affect embryo division timings.

The basis for the age cut-off, <38 years old, was based on data from Liu et al, who reported that Embryoscope time-lapse imaging of polar body 2 extrusion was significantly delayed for women >38 years than for those <35 years and between 35-38 years old (29). Additionally, in PLOS1 in 2017, Grøndahl et al published data showing a tendency toward increased time between cell cleavages with increasing maternal age (46).

Also, we excluded other factors that can contribute to abnormal embryo development. Specifically, we excluded patients with diminished ovarian reserve as there is data that AMH and antral follicle count are comparable predictors of oocytes retrieved and of the number of good-quality embryos available for transfer and freezing (30). Some investigators have found a positive correlation between AMH levels and oocyte and embryo quality (31, 32). We felt that potentially having lower numbers of embryos retrieved and a higher risk of poor embryo development resulting in cycle cancellation could skew the results of those with diminished ovarian reserve. However, we do note since we started our study, studies have been published demonstrating that AMH levels were not predictive of time-lapsed imaging division timings (47, 48)

Along those lines of factors that can predispose to abnormal embryo development, a retrospective cohort study of IVF cycles in the United States between 2009 and 2013 showed that cigarette smoking was associated with higher adjusted odds of cycle cancellation with no embryo transfer (aOR: 1.10; 95% CI: 1.00–1.21) (33). Additionally, in a review of the impact of cigarette smoking on reproduction, Dechanet et al describe how cigarette smoke exposure may induce meiotic spindle disturbances and disordered microfilaments in rodent models (34). They conclude, “Different molecules contained in cigarette smoke have different pro- or/and anti-apoptosis properties, leading to a probable impairment of embryo development…” The mechanisms involved in the effects of smoke on embryo apoptosis or survival remain unclear and must be elucidated.

## Conclusion

In light of our findings, contrary to our hypothesis that division times would be consistently longer in patients with higher BMI categories, it is clear that further studies are warranted to evaluate other potential factors influenced by obesity. Our findings demonstrating a lack of BMI-dependent alteration in embryo division timings, together with our pattern of more rapid T2 corresponding to improved IVF outcomes, suggest a more complex, alternative mechanism by which BMI alters embryo development.
